# Use of Digital Technology for Education: The Future of Collaborative Learning

**DOI:** 10.5704/MOJ.2103.025

**Published:** 2021-03

**Authors:** SHS Lai, CQY Tang

**Affiliations:** 1 Department of Infectious Disease, Tan Tock Seng Hospital, Singapore; 2 Department of Hand and Reconstructive Microsurgery, Tan Tock Seng Hospital, Singapore

Dear editor,

We read with interest the article “COVID-19 in Singapore and Malaysia: Rising to the Challenges of Orthopaedic Practice in an Evolving Pandemic” by Tay *et al*^1^. As highlighted in the article, medical educationist harnessed the powers of technology for medical education and orthopaedic training. To add on to the discussion, we hope to highlight the role of digital learning, and speak from our personal experience with distance learning via digital platform.

In this coronavirus disease 2019 (COVID-19) crisis, we notice a silver lining - a strong push towards digital e-learning, with greater support for distance learning and stronger digital infrastructure. Digital technology has played a crucial role during the pandemic with its unique ability to minimise physical contact and break infection chains while allowing collaboration and learning amongst specialists and residents alike. For instance, with conferences cancellations on an unprecedented scale^2^, many are turning to virtual congress and meetings to foster scientific exchanges. This includes live webinars and digital congress meetings, which paradoxically allow more to attend by reducing time off clinical workload.

The medical world is coming together to train young doctors as well, with free online resources offered by various societies to continue medical education for surgical trainees and residents, such as that by AO Trauma. We speak from our personal experience with distance learning. Before COVID-19 struck, both authors participated in a nine-month course entitled Principles and Practice of Clinical Research organised by Harvard T.H Chan School of Public Health which offers training in clinical research and endeavours to create a global network of clinical researchers^3^. As web remote-access participants, we took part in weekly live lectures, forum discussions, group project, examinations and feedback sessions, with all learning activities being carried out on digital platforms. Use of various online tools such as Google classroom and Ryver forums enabled round-the-clock learning via digital methods. We graduated from the course with a better understanding of the concepts in clinical research, and are currently teaching assistants for the programme.

Others too have reported positive experiences with digital learning, citing examples of anatomical education and surgical training conducted through online tools such as virtual interactive anatomy and simulations^4^. As the medical community adapts to these new working conditions, it is apparent that knowledge sharing may never be the same again^5^.

We firmly believe that COVID-19 will be the impetus towards a future of collaborative learning in the medical field, and we strongly urge more to consider embracing distance and digital learning. It will truly allow knowledge sharing that is no longer bounded by geographical constraints.

## References

[1] Tay K, Kamarul T, Lok WY, Mansor M, Li X, Wong J (2020). COVID-19 in Singapore and Malaysia: Rising to the Challenges of Orthopaedic Practice in an Evolving Pandemic.. Malays Orthop J.

[2] Viglione G (2020). A year without conferences? How the coronavirus pandemic could change research.. Nature..

[3] Harvard T.H Chan School of Public Health. Principles and Practice of Clinical Research. Vol 2020: The President and Fellows of Harvard College. http://https://execed.sph.harvard.edu/principles-and-practice-of-clinical-research.

[4] Moszkowicz D, Duboc H, Dubertret C, Roux D, Bretagnol F (2020). Daily medical education for confined students during coronavirus disease pandemic: A simple videoconference solution.. Clin Anat..

[5] Achakulvisut T, Ruangrong T, Bilgin I, Van Den Bossche S, Wyble B, Goodman DF (2020). Improving on legacy conferences by moving online.. Elife.

